# 

**Published:** 1907-12

**Authors:** 


### The Library of the Bristol Medico-Chirurgical Society.

*The following donations have been received since the publication of the List in September:*

*November 30th,* 1907.

| | |
|---|---|
| The Chicago Pathological Society (1) .. .. | 1 volume. |
| L. M. Griffiths (2) .. .. .. .. .. | 7 volumes. |
| Dr. A. B. Judson (3) .. .. .. .. .. | 1 volume. |
| The Middlesex Hospital (4) .. .. .. .. | 1 ,, |
| The Surgeon-General, United States Army (5) .. | 1 ,, |

### SIXTY-SIXTH LIST OF BOOKS.

The titles of books mentioned in previous lists are not repeated. The figures in brackets refer to the figures after the names of the donors and show by whom the volumes are presented. The books to which no such figures are attached have either been bought from the Library Fund, or received through the *Journal*.

| | | |
|---|---|---|
| **Allbutt and H. D. Rolleston, T. C.** [Eds.] | *A System of Medicine* Vol. III. | 1907 |
| **Berdoe, M.** .. .. | *Essay on the Pudendagra* .. .. .. (2) | 1771 |
| **Bigg, G. S.** .. .. | *Cancer* .. .. .. .. .. .. .. .. .. .. | 1907 |
| **Bourcart et F. Cautru, M.** | *Le Ventre.* Pars II... .. .. .. | 1908 |
| *British Pharmaceutical Codex, The* .. .. .. .. .. .. .. .. .. | | 1907 |
| **Bunge, G. v.** .. | *Text-Book of Organic Chemistry.* (Tr. by R. H. A. Plimmer) .. .. .. .. .. .. | 1907 |
| **Campbell, H.** .. | *Aids to Pathology* .. .. .. .. .. .. | 1908 |
| *Catalogue (Index-) of the Library of the Surgeon-General's Office, United States Army.* 2nd Ser., Vol. XII. (5) | | 1907 |
| **Cautru, M. Bourcart et F.** | *Le Ventre.* Pars II... .. .. .. | 1908 |
| **[Cecil]** .. .. .. .. | *The Stud Farm* .. .. .. (2) New Ed. | 1876 |
| **Collier, R. Hutchison and H. S.** [Eds.] | *An Index of Treatment* | 1907 |

**Corner and H. I. Pinches, E. M.** *The Operations of General Practice* .. .. .. .. .. .. .. .. .. 1907

**Crowe, H. W.** .. *Consumption: Home Treatment and Rules for Living* .. .. .. .. .. [2nd Ed.] 1907

**Cunningham, D. J.** *Manual of Practical Anatomy.* 2 vols. 4th Ed. 1907

**Dampier-Bennett, A. G.** *Physical Methods in the Treatment of Heart Disease* .. .. .. .. .. .. .. 1907

**Dore, M. Morris and S. E.** *Light and X-ray Treatment of Skin Diseases* .. .. .. .. .. .. .. .. 1907

*Encyclopedia and Dictionary of Medicine and Surgery.* Green's Vols. V., VI. [1907]

**Esmarch, F.** .. .. *First Aid to the Injured.* (Tr. by H.R.H. Princess Christian) .. .. 7th Ed. 1907

**Fernie, W. T.** .. *Precious Stones (Curative)* .. .. .. .. .. 1907

**Goodall, G. H. Savage and E.** *Insanity and Allied Neuroses* New [3rd] Ed. 1907

**Harman, N. B.** .. *Preventable Blindness* .. .. .. .. .. .. 1907

**Hart, A. H.** .. .. *Some Successful Prescriptions* .. .. .. 1907

**Hughes, E. L.** .. *Squint and Ocular Paralysis* .. .. .. 1907

**Hutchison and H. S. Collier, R.** [Eds.] *An Index of Treatment* .. .. .. .. .. .. .. .. .. .. 1907

**Jones, R.** .. .. *Mental and Sick Nursing* .. .. .. .. 1907

**Judson, A. B.** .. *Growth and Deformity* .. .. .. (3) 1905

**Kenwood, L. C. Parkes and H. R.** *Hygiene and Public Health* 3rd Ed. 1907

**M'Vail, J. C.** .. *The Prevention of Infectious Diseases* .. 1907

**Manson, Sir P.** .. *Tropical Diseases* .. .. .. .. .. 4th Ed. 1907

**Micholitsch, E. Wertheim and T.** *The Technique of Vagino-Peritoneal Operations.* (Tr. by C. Lockyer) 1907

**Morris and S. E. Dore, M.** *Light and X-ray Treatment of Skin Diseases* .. .. .. .. .. .. .. .. 1907

**Muir and J. Ritchie, R.** *Manual of Bacteriology* .. .. 4th Ed. 1907

**Nelson, J.** .. .. *The Government of Children* (2) 2nd Ed. 1756

**Newman, D.** .. .. *Movable Kidney* .. .. .. .. .. .. .. 1907

**Nichols, J. B.** .. *Diet in Typhoid Fever* .. .. .. .. .. 1907

**Noorden, C. von** .. *Metabolism and Practical Medicine.* (Ed. by I. W. Hall) Vol. III. .. .. .. .. .. 1907

**Parkes and H. R. Kenwood, L. C.** *Hygiene and Public Health* 3rd Ed. 1907

**Parsons, J. H.** .. *Diseases of the Eye* .. .. .. .. .. .. 1907

**Partridge, W.** .. *The Bacteriological Examination of Disinfectants* .. .. .. .. .. .. .. .. 1907

**Paton, D. N.** .. *Essentials of Human Physiology* 3rd Ed. 1907

**Pinches, E. M. Corner and H. I.** *The Operations of General Practice* 1907

**Ritchie, R. Muir and J.** *Manual of Bacteriology* .. 4th Ed. 1907

**Robson, A. W. M.** *Cancer of the Stomach* .. .. .. .. .. .. 1907

**Rolleston, T. C. Allbutt and H. D.** [Eds.] *A System of Medicine* Vol. III. 1907

**Russell, Hon. R.** .. *The Reduction of Cancer* .. .. .. .. .. 1907

**Sajous, C. E. de M.** *The Internal Secretions.* Vol. II. .. .. 1907

**Saundby, R.** .. .. *Medical Ethics* .. .. .. .. .. 2nd Ed. 1907
**Savage and E. Goodall, G. H.** *Insanity and Allied Neuroses* New [3rd] Ed. 1907
**Schrötter, L. von** .. *Hygiene of the Lung.* (Tr. by H. W. Armitt) 1907
**Stephenson, S.** .. *Ophthalmia Neonatorum* .. .. .. .. 1907
**Taylor, S.** .. .. *On Acute Pneumonia* .. .. .. .. .. .. 1907
**Treves, Sir F.** .. *Surgical Applied Anatomy* (Revised by A. Keith) .. .. .. .. .. 5th Ed. 1907
**Turner, W. A.** .. *Epilepsy* .. .. .. .. .. .. .. .. 1907
**Wertheim and T. Micholitsch, E.** *The Technique of Vagino-Peritoneal Operations.* (Tr. by C. Lockyer) 1907
**Wilson, T.** .. *Pelvic Inflammations in the Female* .. .. 1907
**Wynter, W. E.** .. *Minor Medicine* .. .. .. .. .. .. .. 1907

### TRANSACTIONS, REPORTS, JOURNALS, &c.

American Journal of Obstetrics, The .. .. .. Vol. LV. 1907
American Journal of the Medical Sciences, The Vol. CXXXIII. 1907
American Pediatric Society, Transactions of the Vol. XVIII. 1907
Archives of the Middlesex Hospital .. .. .. (4) Vol. XI. 1907
Boston Medical and Surgical Journal, The .. .. Vol. CLVI. 1907
Bristol Health Report for 1906 .. .. .. .. .. .. .. 1907
British Gynæcological Journal, The .. .. Vol. XXII. 1906–07
Chicago Pathological Society, Transactions of the (1) Vol. VI. 1906
Clinical Journal, The .. .. .. .. .. .. .. .. Vol. XXX. 1907
Glasgow Medical Journal, The .. .. .. .. Vol. LXVII. 1907
Henry Phipps Institute, Third Annual Report of the .. .. .. 1907
Hospital, The .. .. .. .. .. .. .. Vols. XLI., XLII. 1907
Journal of Medical Research, The .. .. .. .. Vol. XVI. 1907
Journal of Mental Pathology, The .. .. .. .. Vol. VII. [1907]
Journal of Obstetrics and Gynæcology, The .. .. Vol. XI. 1907
Journal of the American Medical Association, The Vol. XLVIII. 1907
Library Association Record, The .. .. .. .. Vol. VII. 1905
Library World, The .. .. .. .. .. .. .. .. Vol. IX. 1906–07
Medical Chronicle, The .. .. .. .. .. N.S., Vol. XII. 1906–07
Medical Press and Circular, The .. .. .. [Vol. CXXXIV.] 1907
Medical Record .. .. .. .. .. .. .. .. .. Vol. LXXI. 1907
Odontological Society of Great Britain, Transactions of the Vol. XXXIX. 1907
Practitioner, The .. .. .. .. .. .. .. [Vol. LXXVIII.] 1907
Progressive Medicine .. .. .. .. .. .. .. Vol. III. 1907
Royal Academy of Medicine in Ireland, Transactions of the Vol. XXV. 1907
Scottish Medical and Surgical Journal, The .. Vol. XX. 1907
Sociedad de Beneficencia de Buenos Aires—Memoria del 1906 .. 1907
Univ. of Penna. Medical Bulletin .. .. .. .. Vol. XIX. 1907

### Local Medical Notes.

**University College, Bristol.**—Examination Results :—

M.B., B.S. Lond.—A. J. M. Wright (Distinguished in Medicine and Surgery).

W. S. V. Stock, M.B., B.S. Lond., and S. C. Hayman, M.R.C.S., L.R.C.P., have passed the final examination for the Fellowship of the Royal College of Surgeons, England.

M.D. Durh.—H. T. S. Aveline, M.R.C.S.

M.R.C.P. Lond.—George Basil Price, M.D. Lond.

Conjoint Board.—*Practical Pharmacy:* L. C. Watkins-Baker, T. E. Ashley. *Anatomy and Physiology:* B. C. Eskell, T. S. Rippon. *Surgery:* C. Clarke, P. S. Tomlinson, E. V. Connellan. *Midwifery:* E. R. Sircom.

L.S.A. Lond.—*Medicine, Section II.:* E. V. Connellan.

L.D.S. Lond.—*Chemistry:* G. Smith. *Final Examination:* Cuthbert G. Plumley.

**Bristol Royal Infirmary.**—A. Lewin Sheppard, M.B., B.S. Durh., has been appointed Senior Resident Officer and House Surgeon, and A. W. Falconer, M.D. Aberd., House Physician.

**Bristol General Hospital.**—The following appointments have been recently made:—*House Physician:* C. S. Rivington, M.R.C.S., L.R.C.P. *House Surgeon:* J. W. J. Willcox, M.R.C.S., L.R.C.P. *Casualty House Surgeon:* A. E. Iles, M.R.C.S., L.R.C.P. *Assistant House Physician:* W. Bruce Low, M.B., B.Ch. Edin.

**Long Fox Lecture.**—The next Long Fox Lecture will be delivered by Dr. Watson Williams, on Thursday, January 16th, 1908, at 4.15 p.m. in the Medical Library, University College, Bristol. Subject: "*Suppurative Disease in the Nose and Ear, with special reference to newer methods in Diagnosis and Treatment.*"

### BRISTOL.

**Consultations at the Bristol Workhouses.**—At a recent meeting the Bristol Board of Guardians considered a report of the hospital committee, which contained a recommendation to appoint a consultant to be availaible in case of difficulty occurring in the treatment of patients in the Infirmary wards at either of the two workhouses, or where the workhouse medical officer would like to have the advantage of a second opinion. In the discussion of the report it was stated that there were no suitable operating theatres at the workhouse infirmaries, and that it was the custom to send patients for operation to the Royal Infirmary.

### BATH.

**Bath Royal United Hospital.**—Thanks to the efforts of the Mayor of Bath (Mr. S. W. Bush), this institution is now free from debt. His Worship, at the outset of his mayoralty, announced that his endeavour would be to free the hospital from its debt of £6,124, and we heartily congratulate him upon his success.

**Bath Royal Mineral Water Hospital.**—The governors of this hospital have decided to decrease the number of beds from 155 to 150 from lack of funds, but the Mayor of Bath is making an effort to secure increased support, so that this retrograde step shall not be necessary. It is pointed out that of the present 149 in-patients only one belongs to Bath. It is claimed, therefore, that the hospital is national in its operations, and justly appeals for national help.

**Winsley Sanatorium.**—Alfred Lewthwaite, M.B. Lond., has been appointed Resident Medical Officer to this institution, vice E. D. Townroe, resigned.

### INDEX.

(R.) = Review.

Abdominal aorta, Aneurysm of the—Dr. F. P. Nunneley (R.), 353.
Acetone bodies, The, 59.
Adami, Dr. J. G.—Inflammation (R.), 355.
American Pediatric Society, Transactions of the (R.), 172
American Surgery in 1906, Glimpses of—C. H. Whiteford (R.), 264.
Anæmia, Pernicious, 239.
Anæsthetic poisoning, Delayed, 156.
Anæsthetics, Guide to—Dr. T. D. Luke (R.), 364.
Anæsthetics, their uses and administration—Dr. D. W. Buxton (R.), 363; Dr. F. W. Hewitt (R.), 362.
Anatomy: Manual of—Dr. A. M. Buchanan (R.), 251, 354; Textbook of—Dr. D. J. Cunningham (R.), 168.
Aneurysm of the abdominal aorta—Dr. F. P. Nunneley (R.), 353.
Angioma of the liver, Acquired—Dr. A. L. Sheppard, 46.
An Imperial Dispensatory: The British Pharmaceutical Codex (R), 358.
Antenatal pathology and hygiene: the embryo—Dr. J. W. Ballantyne (R.), 254.
Aorta, Compression of, in treatment of post-partum hemorrhage—Dr. F. P. Elliott, 121, 310.
Aphonia, A suggested treatment for functional—Dr. C. P. Crouch, 214.
Atheroma, Experimental, 342.
Atkinson, Dr. S. B.—Golden rules of medical evidence (R.), 364.
Auscultation and percussion—Dr. S. Gee (R.), 362.

Bacterial origin of general paresis, 348.
Bacteriology, Applied—C. G. Moor and R. T. Hewlett (R.), 171.
Ball, Dr. J. B.—A handbook of diseases of the nose and pharynx (R.), 173.
Ballantyne, Dr. J. W.—Antenatal pathology and hygiene: the embryo (R.), 254.
Banti's disease with complications—Dr. R. Roxburgh, 286.
Beasley's book of prescriptions (R.), 261.
Beddoe, Presentation of portrait of Dr. John, 288.
Bier's method of producing passive hyperæmia, 61.
Bigg, H.—Spinal curvatures (R.), 264.
Biographic clinics—Dr. G. M. Gould (R.), 255.
Blackham, R. J.—The care of children (R.), 75.
Bland-Sutton, J.—Gallstones and diseases of the bile-ducts (R.), 170; Tumours, innocent and malignant (R.), 361.
Blood changes in general sarcoma—Dr. F. G. Bushnell, 321.
Blood-glands as pathogenic factors in the production of diabetes, 57.
Blood pressure, Studies in—Dr. G. Oliver (R.), 73.
Breast, Cancer of the—W. S. Handley (R.), 253.
Breast, Excision of, for carcinoma, 344.
Brend, Dr. W. A.—A handbook of medical jurisprudence and toxicology (R.), 261.
Bristol General Hospital, Description of new isolation block at, 93.
Bristol Medico-Chirurgical Society, 87, 185, 285, 382.
Bristol, Public health in, 1906–7—Dr. D. S. Davies, 336.
Bristol Royal Infirmary, Proposed new rules, 272, 368.
British journal of tuberculosis (R.), 263.
Brown and W. T. Ritchie, Drs. J. J. G.—Medical diagnosis (R.), 262.
Brunton, Sir L.—Collected papers on circulation and respiration (R.), 171.

Buchanan, Dr. A. M.—Manual of anatomy (R.), 251, 354.
Bullen, F. St. J.—Report on psychiatry, 348.
Bush and Dr. J. A. Nixon, J. P.—A case of filariasis, 218.
Bushnell, Dr. F. G.—A case of generalised sarcoma, with blood changes, 321.
Buxton, Dr. D. W.—Anæsthetics, their uses and administration (R.), 363.

Calder, Dr. A. B.—Lectures on midwifery for midwives (R.), 171.
Campbell, Dr. H.—On treatment (R.), 260.
Cancer of the breast—W. S. Handley (R.), 253.
Carcinoma of breast, 344.
Carter, Dr. A. H.—Elements of practical medicine (R.), 262.
Carwardine, T.—Report on surgery, 243.
Catalogue (Index-) of the library of the Surgeon-General's Office, United States Army (R.), 270.
Catalogue of the pathological museum of the Manchester University—Dr. J. L. Smith (R.), 364.
Cataract extraction, Healing of corneal wound in, 249.
Cathcart, Dr. C. W.—The essential similarity of innocent and malignant tumours (R.), 254.
Cerebral lesions in pregnancy and parturition—Dr. W. C. Swayne, 209.
Cerebro-spinal fever—Drs. D. S. Davies and I. W. Hall, 14.
Cerebro-spinal fever, 82.
Cerebro-spinal meningitis, The etiology and diagnosis of—Dr. A. W. Taves (R.), 262.
Chapman, Dr. W. L.—The sequelæ of gonorrhœa in both sexes (R.), 265.
Charles, Dr. J. R.—Report on medicine, 57.
Chest, The Röntgen rays in the diagnosis of diseases of the—Drs. H. Walsham and C. H. Orton (R.), 267.
Children, Aids to the treatment of diseases of—Dr. J. McCaw (R.), 365.
Children, The care of—R. J. Blackham (R.), 75.
Circulation, Collected papers on—Sir L. Brunton (R.), 171.
Clarke, Dr. J. M.—Cases illustrating the more unusual complications of pneumonia, 89, 108; Report on medicine, 341; Treatment of Graves's disease by anti-thyreoid serum and by X-rays, 201.
Climatotherapy and balneotherapy—Sir H. and Dr. F. P. Weber (R.), 266.
Cod liver oil and tuberculosis—Dr. J. W. Wells (R.), 271.
Colour phenomena—J. W. Lovibond (R.), 72.
Consciousness, Double—Dr. F. H. Edgeworth, 186.
Convulsions, 67.
Coombs, Dr. C.—Rheumatic carditis in childhood, 193.
Cooper, R. H.—The uses of X-rays in general practice (R.), 268.
Corneal wound in cataract extraction, Healing of, 249.
Cossham Memorial Hospital, 179, 190.
Cotton, Dr. W.—Proportional representation and the comparison of radiographs, 326.
Crichton-Browne, Sir J.—The prevention of senility, and a sanitary outlook (R.), 360.
Crook, Dr. H. E.—High frequency currents (R.), 257.
Crouch, Dr. C. P.—A suggested treatment for functional aphonia, 214.
Cuff, I. Stewart and Dr. H. E.—Practical nursing (R.), 175.
Cunningham, Dr. D. J. [Ed.]—Textbook of anatomy (R.), 168.

Dalgado, Dr. D. G.—The climate of Lisbon, Mont Estoril and Cintra (R.), 173.
David, Dr. M.—Grundriss der orthopadischen chirurgie (R.), 265.
Davies, Dr. D. S.—Public health in Bristol, 1906–7, 336.
Davies and I. W. Hall, Drs. D. S.—Cerebro-spinal fever, 14.
Davos as health resort (R.), 173.
Deflections and spurs of the nasal septum, Operations for—Dr. P. W. Williams, 21.
Dental caries, On—Dr. J. S. Wallace (R.), 175.
Diabetes, Blood-glands as pathogenic factors in, 57.
Digestive system, Treatment of diseases of the—Dr. R. Saundby (R.), 73.

Dowse, Dr. T. S.—Lectures on massage and electricity (R.), 268.

Editorial notes, 76, 177, 272, 368.
Edmunds, W.—Sound and rhythm (R.), 174.
Electricity, medical—Dr. T. S. Dowse (R.), 268 ; Dr. H. L. Jones (R.), 266.
Elliott, Dr. F. P.—The value of compression of the aorta in the treatment of post-partum hemorrhage, 121, 310.
Encyclopedia and dictionary of medicine and surgery (R.), 259.
Epidemiological Society of London, Transactions of the (R.), 269.
Estlander's operation for empyema —Dr. H. S. Ballance, 285.
Evidence, Golden rules of medical— Dr. S. B. Atkinson (R.), 364.
Eye, Diseases of the—Dr. C. H. May and C. Worth (R.), 172.
Eye, Syphilitic affections of the, 247.

Fæces, 68.
Filariasis, Case of—J. P. Bush and Dr. J. A. Nixon, 218.
Food-fads and faddists, 368.
Foster, Sir Michael, 81.
Free, John, 276.

Gallstones and diseases of the bile-ducts—J. Bland-Sutton (R.), 170.
Gee, Dr. S.—Auscultation and percussion (R.), 362.
Gonorrhœa, The sequelæ of—Dr. W. L. Chapman (R.), 265.
Gould, Dr. G. M.—Biographic clinics (R.), 255.
Gout—Dr. A. P. Luff (R.), 260.
Graves's disease treated by antithyreoid serum and by X-rays— Dr. J. M. Clarke, 201.
Griffiths, L. M.—The Medical Reading Society, Bristol, 222.
Groves, E. W. H.—Report on surgery, 61 ; Some remarks on spinal anæsthesia, as based upon the personal observation of thirty cases, 305.
Guy's hospital reports (R.), 271.

Hall, Dr. I. W.—Report on medicine, 236; Report on pathology, 67.
Hall, Drs. D. S. Davies and I. W.— Cerebro-spinal fever, 14.
Handley, W. S.—Cancer of the breast and its operative treatment (R.), 253.
Herman, Dr. G. E.—Diseases of women (R.), 366 ; First lines in midwifery (R.), 362.
Hernia, Retro-peritoneal—B. G. A. Moynihan (R.), 75.
Herringham, Dr. W. P.—On physical training in schools (R.), 174.
Hewitt, Dr. F. W.—Anæsthetics and their administration (R.), 362.
Hewlett, C. G. Moor and R. T. — Applied bacteriology (R.), 171.
High frequency currents—Dr. H. E. Crook (R.), 257.
Horsfall, T. C.—The influence on national life of military training in schools (R.), 174.
Hyperæmia, Bier's method of producing passive, 61.
Hyperleucocytosis, 350.
Hypertension, 153.

Infectious diseases, Nursing of— Dr. F. J. Woollacott (R.), 176.
Inflammation—Dr. J. G. Adami (R.), 355.
Internal organs, Indications for operation in disease of the— Dr. H. Schlesinger (R.), 70.
Intestine, Two cases of ruptured— H. F. Mole, 38.

Jones, Dr. H. L.—Medical electricity (R.), 266.

Kendall, Obituary notice of Herbert W., 90.
Klein, Dr. E.—Studies in the bacteriology and etiology of oriental plague (R.), 361.
Knee-joint, A new method of fixation in excision of the—E. W. H. Groves, 88.

Latham, Dr. A.—Pulmonary consumption (R.), 364.
Library of the Bristol Medico-Chirurgical Society, 85, 182, 283, 379.
Lisbon, The climate of—Dr. D. G. Dalgado (R.), 173.
Liver, Acquired angioma of the— Dr. A. L. Sheppard, 46.
Local medical notes, 93, 187, 287, 383.
Lovibond, J. W.—An introduction to the study of colour phenomena (R.), 72.

Luff, Dr. A. P.—Gout (R.), 260.
Luke, Dr. T. D.—Guide to anæsthetics (R.), 364.
Lumbar puncture as an aid to diagnosis, 351.
Lupus vulgaris, Eighty cases of—Dr. W. K. Wills, 127.

McCaw, Dr. J.—Aids to the treatment of diseases of children (R.), 365.
McCrudden, F. H.—Uric acid: chemistry, physiology and pathology (R.), 74.
M'Gregor, Dr. A. N.—A system of surgical nursing (R.), 175.
McKisack, Dr. H. L.—A dictionary of medical diagnosis (R.), 259.
Marshall, Dr. C. F.—Syphilology and venereal disease (R.), 70.
Martindale and Dr. W. W. Westcott, W.—The extra pharmacopœia (R.), 75.
Massage, Lectures on—Dr. T. S. Dowse (R.), 268.
Massage, Lessons on—M. D. Palmer (R.), 268.
May and C. Worth, Dr. C. H.—A manual of diseases of the eye (R.), 172.
Medical diagnosis—Drs. J. J. G. Brown and W. T. Ritchie (R.), 262.
Medical diagnosis, A dictionary of—Dr. H. L. McKisack (R.), 259.
Medical diagnosis, Aids to—Dr. A. Whiting (R.), 353.
Medical jurisprudence, A handbook of—Dr. W. A. Brend (R.), 261.
Medical Reading Society, Bristol—L. M. Griffiths, 222.
Medicine, Manual of—Dr. T. K. Monro (R.), 73.
Medicine, Practical—Dr. A. H. Carter (R.), 262.
Medicine, Reports on—Dr. J. R. Charles, 57; Dr. J. M. Clarke, 341; Dr. I. W. Hall, 236; Dr. G. Parker, 152.
Midwifery, First lines in—Dr. G. E. Herman (R.), 362.
Midwifery for midwives, Lectures on—Dr. A. B. Calder (R.), 171.
Miles, A. Thomson and A.—Manual of surgery (R.), 263.
Military training in schools, Influence on national life of—T. C. Horsfall (R.), 174.
Milk supply of schools, Inquiry concerning the—Dr. C. E. Shelly (R.), 172.
Minor maladies and their treatment—Dr. L. Williams (R.), 74.
Mole, H. F.—Two cases of ruptured intestine, 38.
Monkeys, Tumours and tubercle in—W. R. Williams, 148.
Monro, Dr. T. K.—Manual of medicine (R.), 73.
Moor and R. T. Hewlett, C. G.—Applied bacteriology (R.), 171.
Morton, C. A.—Report on surgery, 156.
Moynihan, B. G. A.—On retroperitoneal hernia (R.), 75.
Mummery, Dr. P. L.—The sigmoidoscope (R.), 263.
Muthu, Dr. D. J. C.—The sanatorium treatment of pulmonary tuberculosis—is it a success? 50.
Myeolomata and albumosuria, Multiple—Dr. J. R. Charles, 89.

Narcolepsy, A case of—Dr. B. M. H. Rogers, 87, 144.
Nasal septum, Operations for deflections and spurs of the—Dr. P. W. Williams, 21.
Neerlandicorum de arte medica, Opuscula selecta (R.), 361.
Nixon, J. P. Bush and Dr. J. A.—A case of filariasis, 218.
Nose and pharynx, Diseases of the—Dr. J. B. Ball (R.), 173.
Nose and throat, Treatment of diseases of the—C. A. Parker (R.), 69.
Nunneley, Dr. F. P.—Aneurysm of the abdominal aorta (R.), 353.
Nursing, Practical—I. Stewart and Dr. H. E. Cuff (R.), 175.
Nutritional disorders of infancy, Treatment of—Dr. R. Vincent (R.), 258.

Obituary, 90, 97.
Obstetrics, Report on—Dr. W. C. Swayne, 162.
Ogilvy, Dr. A.—Report on ophthalmology, 247.
Oliver, Dr. G.—Studies in blood pressure (R.), 73.
Ophthalmology, Report on—Dr. A. Ogilvy, 247.
Orthopadischen chirurgie, Grundrissder—Dr. M. David (R.), 265.
Orton, Drs. H. Walsham and C. H.—The Röntgen rays in the diagnosis of diseases of the chest (R.), 267.

Page, F. J. M.—Elements of physics for medical students (R.), 357.
Palmer, M. D.—Lessons on massage (R.), 268.
Paresis, Bacterial origin of general, 348.
Paresis, Change of type in general, 352.
Parker, C. A.—A guide to the diseases of the nose and throat, and their treatment (R.), 69.
Parker, Dr. G.—Report on medicine, 152.
Pathogenic organisms, 83.
Pathogenic streptococci, The, 236.
Pathological demonstrations, 96.
Pathology, Report on—Dr. I. W. Hall, 67.
Peritonitis, Treatment of general, 243.
Pharmacopœia, The extra—W. Martindale and Dr. W. Westcott (R.), 75.
Philadelphia hospital reports (R.), 263.
Philippine journal of science, The (R.), 174.
Physical training in schools, On—Dr. W. P. Herringham (R.), 174.
Physics, Elements of—F. J. M. Page (R.), 357.
Plague, Bacteriology and etiology of oriental—Dr. E. Klein (R.), 361.
Pleural effusions, 152.
Pneumonia, Unusual complications of—Dr. J. M. Clarke, 89, 108.
Post-partum hemorrhage, 162.
Post-partum hemorrhage, The value of compression of the aorta in—Dr. F. P. Elliott, 121, 310.
Pregnancy and parturition, Cerebral lesions in—Dr. W. C. Swayne, 209.
Prescriptions, Beasley's book of (R.), 261.
Prognosis—Dr. P. H. Pye-Smith, 1.
Psychiatry, Report on—F. St. J. Bullen, 348.
Pulmonary consumption—Dr. A. Latham (R.), 364.
Pulmonary tuberculosis, The sanatorium treatment of—Dr. D. J. C. Muthu, 50.
Pye-Smith, Dr. P. H.—Prognosis, 1,

Radiographs, Proportional representation and the comparison of —Dr. W. Cotton, 326.
Reinhardt, Dr. C.—Some notes on styracol, 54.
Renal anasarca, Chloride deprivation in the treatment of, 343.
Respiration, Collected papers on circulation and—Sir L. Brunton (R.), 171.
Retro-peritoneal hernia—B. G. A. Moynihan (R.), 75.
Rheumatic carditis in childhood—Dr. C. Coombs, 193.
Ritchie, Drs. J. J. G. Brown and W. T.—Medical diagnosis (R.), 262.
Röntgen rays in the diagnosis of diseases of the chest—Drs. H. Walsham and C. H. Orton (R.), 267.
Rogers, Dr. B. M. H.—A case of narcolepsy, 87, 144.
Ruptured intestine, Two cases of—H. F. Mole, 38.

Sage-femme et de la garde, Le livre de la—Dr. R. de Seigneux (R.), 176.
St. Bartholomew's hospital reports (R.), 365.
Sanatorium treatment of pulmonary tuberculosis—Dr. D. J. C. Muthu, 50.
Sanitary outlook, A—Sir J. Crichton-Browne (R.), 360.
Sarcoma, Case of generalised, with blood changes—Dr. F. G. Bushnell, 321.
Saundby, Dr. R.—The treatment of diseases of the digestive system (R.), 73.
Savage, Dr. W. G.—The bacteriological examination of water supplies (R.), 355.
Schlesinger, Dr. H.—Indications for operation in disease of the internal organs (R.), 70.
Seigneux, Dr. R. de—Le livre de la sage-femme et de la garde (R.), 176.
Senility, The prevention of—Sir J. Crichton-Browne (R.), 360.
Sheen, Obituary notice of Alfred, 90.
Shelly, Dr. C. E.—A preliminary inquiry concerning the milk supply of schools (R.), 172.
Sheppard, Dr. A. L.—Acquired angioma of the liver, 46.
Sick, Preparations for the:
Adrenalin and chloretone ointment, 376.
Adrenalin and eucaine tablets, 281.
Alaxa, 181

Sick, Preparations for the:
Anæsthetics, "Wellcome" brand, 279.
Aspirin tablets, 283.
Black wash tablets, 376.
Claroma, 283.
Cholelith pill, 376.
Colalin, 83.
Colalin laxative, 83.
Egmol, 376.
"Elixoid" formates compound, 278.
"Elixoid" pine tar compound, 181.
Ernutin, 279.
Formamint tablets, 83.
Formidine, 281, 375.
Grape nuts, 282.
Helmitol tablets, 283.
Heroin hydrochl. tablets, 283.
Iodalbin, 281.
Kuhn's suction mask, 374.
Manhu diabetic foods, 378.
Miol, 378.
Nizin, 182.
Nutritive liquid peptone with creosote, 280.
Pertussin, 282.
Petroleum emulsion, 376.
Phenofax, 84.
Phenol-phthalein compound, 280.
Phytin preparations, 377.
Quinine acetyl-salicylate, 279.
Soloid—Eosin-azur, 85.
Somatose, Liquid, 83.
Tabloids:
Calcium iodo-ricinoleate, 377.
Calcium lactate, 84.
Carbolic acid and slippery elm, 279.
Gingamint, 279.
Guaiacol camphorate, 84.
Pectoral pastilles, 84.
Slippery elm, 279.
Tannigen, 282.
Tannigen tablets, 283.
Theinhardt's food for infants, 377.
Tilia, 282.
Trional tablets, 283.
Triple glycerophosphates with nuclein, 280.
Veronal tablets, 283.
Sigmoidoscope, The—Dr. P. L. Mummery (R.), 263.
Skerritt, Obituary notice of Edward Markham, 97.
Small-pox, Selected essays on syphilis and (R.), 260.
Smith, Dr. J. L.—Catalogue of the pathological museum of the Manchester University (R.), 364.
Smithsonian institution report (R.), 367.
Society, Bristol Medico-Chirurgical, 87, 185, 285, 382.
Society for the study of disease in children, Reports of the (R.), 270.
Sound and rhythm—W. Edmunds (R.), 174.
Spinal anæsthesia, 159.
Spinal anæsthesia, Some remarks on —E. W. H. Groves, 305.
Spinal curvatures—H. Bigg (R.), 264.
Splenomegalic cirrhosis in a child aged seven years, Concluding notes on a case of—Dr. E. C. Williams, 43, 88.
Stacpoole, F. — Women's health, and how to take care of it (R.), 266.
Stewart and Dr. H. E. Cuff, I.—Practical nursing (R.), 175.
Styracol, Some notes on—Dr. C. Reinhardt, 54.
Surgery, Manual of—A. Thomson and A. Miles (R.), 263.
Surgery, Reports on—T. Carwardine, 243; E. W. H. Groves, 61; C. A. Morton, 156; J. Swain, 344.
Surgical nursing, A system of—Dr. A. N. M'Gregor (R.), 175.
Swain, Dr. J.—Report on surgery, 344.
Swayne, Dr. W. C.—Cerebral lesions in pregnancy and parturition, 209; Report on obstetrics, 162.
Syphilis—Dr. H. Waldo, 289.
Syphilis and small-pox, Selected essays on (R.), 260.
Syphilitic affections of the eye, 247.
Syphilology and venereal disease—Dr. C. F. Marshall (R.), 70.

Taste-fibres, Course of, 341.
Taves, Dr. A. W.—The etiology and diagnosis of epidemic cerebro-spinal meningitis (R.), 262.
Thomson and A. Miles, A.—Manual of surgery (R.), 263.
Thyreoid (anti-) serum, Treatment of Graves's disease by—Dr. J. M. Clarke, 201.
Treatment, On—Dr. H. Campbell (R.), 260.
Tubercle in monkeys, Tumours and —W. R. Williams, 149.
Tuberculosis, Infection in, 69.

Tumours and tubercle in monkeys —W. R. Williams, 149.
Tumours, innocent and malignant—J. Bland-Sutton (R.), 361.
Tumours, The essential similarity of innocent and malignant—Dr. C. W. Cathcart (R.), 254.
Tumours, Trauma as a cause of, 68.
Typhoid, The diagnosis of, 155.

University College, Bristol, Examination successes, 93, 187, 287, 383; Incorporation of medical school with, 93; Medical prize distribution, 373.
University College Colston Society, 76.
University for Bristol, Professor Sadler on a, 78.
University ideal, The, 78.
Uric acid—F. H. McCrudden (R.), 74.

Vincent, Dr. R.—Clinical studies in the treatment of nutritional disorders of infancy (R.), 258.

Waldo, Dr. H.—Syphilis, 289.
Wallace, Dr. J. S.—On the cause and prevention of dental caries (R.), 175.
Walsham and C. H. Orton, Drs. H. —The Röntgen rays in the diagnosis of diseases of the chest (R.), 267.
Walter, Capt. A. E.—X-rays in general practice (R.), 268.
Water supplies, The bacteriological examination of—Dr. W. G. Savage (R.), 355.
Weber, Sir H. and Dr. F. P.—Climatotherapy and balneotherapy (R.), 266.
Wellcome's photographic exposure record and diary (R.), 270.
Wells, Dr. J. W.—The influence of cod liver oil on tuberculosis (R.), 271.
Westcott, W. Martindale and Dr. W. —The extra pharmacopœia (R.), 75.
Whiteford, C. H.—Glimpses of American surgery in 1906 (R.), 264.
Whiting, Dr. A.—Aids to medical diagnosis (R.), 353.
Williams, Dr. E. C.—Concluding notes on a case of splenomegalic cirrhosis in a child aged seven years, 43, 88.
Williams, Dr. L.—Minor maladies and their treatment (R.), 74.
Williams, Dr. P. W.—Operations for deflections and spurs of the nasal septum, with special reference to sub-mucous resection, 21.
Williams, W. R.—Tumours and tubercle in monkeys, 149.
Wills, Dr. W. K.—Eighty cases of lupus vulgaris, 127.
Winsley sanatorium, 372.
Women, Diseases of—Dr. G. E. Herman (R.), 366.
Women's health, and how to take care of it—F. Stacpoole (R.), 266.
Woollacott, Dr. F. J.—Lectures upon the nursing of infectious diseases (R.), 176.
Workmen's compensation act, The, 188.
Worth, Dr. C. H. May and C.—A manual of diseases of the eye (R.), 172.

X-rays in general practice—R. H. Cooper (R.), 268; Capt. A. E. Walter (R.), 268.
X-rays, Treatment of Graves's disease by—Dr. J. M. Clarke, 201.

J. W. Arrowsmith, Printer, Quay Street, Bristol.

